# Supplementary material for: TPX2 lactylation is required for the cell cycle regulation and hepatocellular carcinoma progression
Source: Life Sci Alliance. 2025 Mar 19;8(6):e202402978. doi: 10.26508/lsa.202402978 (PMC11924114; doi:10.26508/lsa.202402978)
Supplement: Supplementary file 2 [file LSA-2024-02978_SdataF2.pdf]

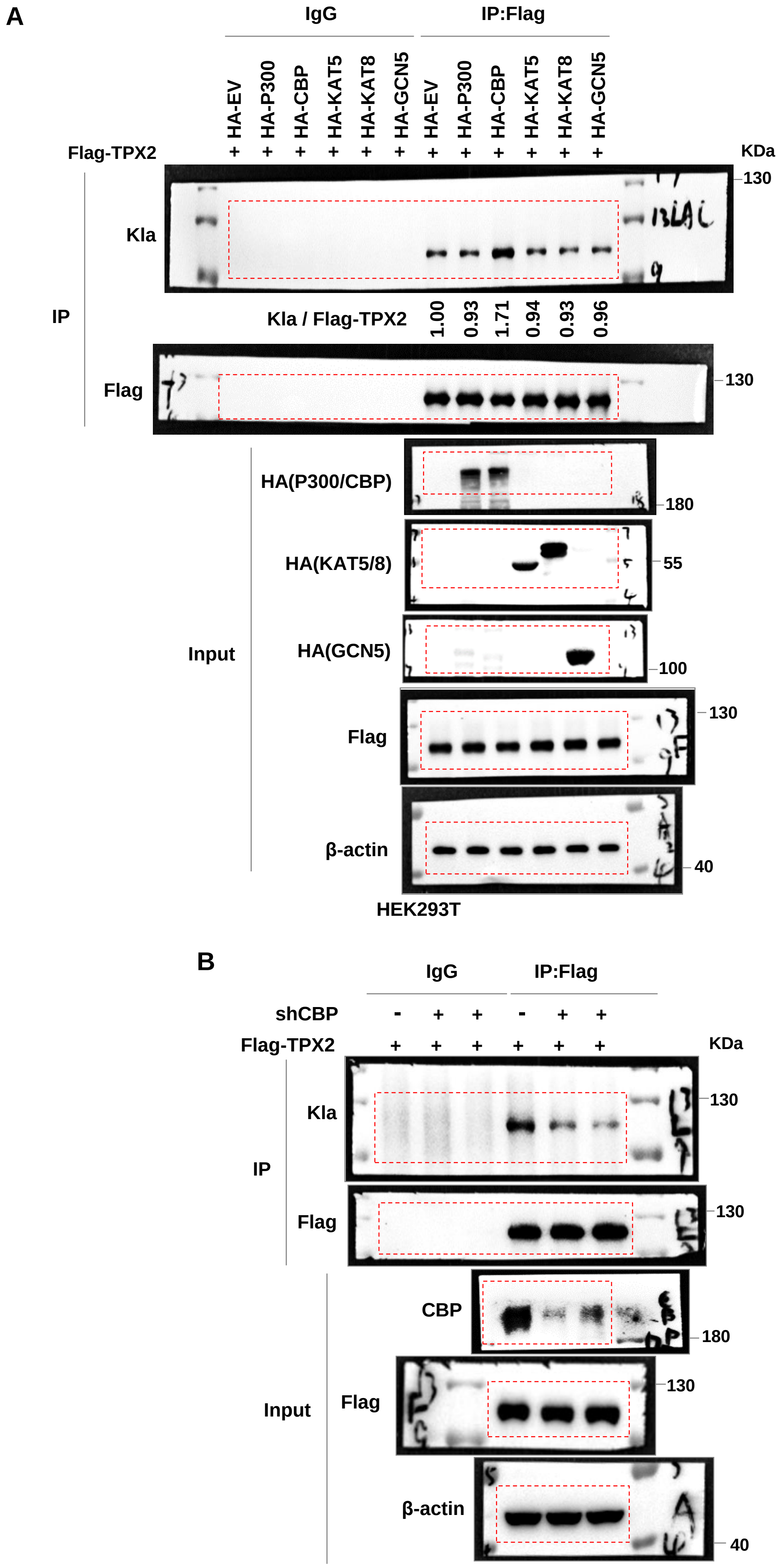

Figure 2. TPX2 is lactylated by CBP and delactylated by HDAC1.

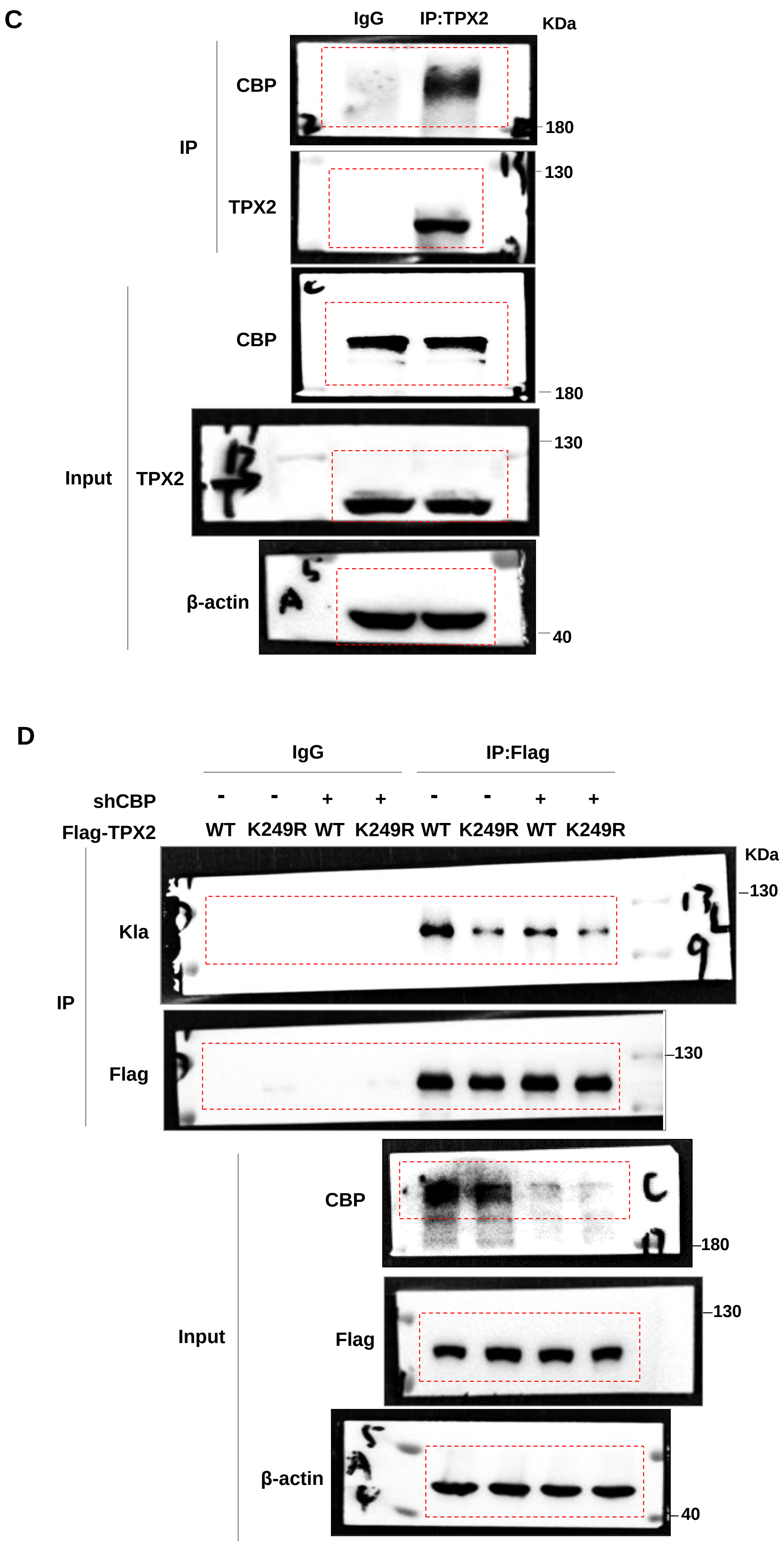

Figure 2. TPX2 is lactylated by CBP and delactylated by HDAC1.

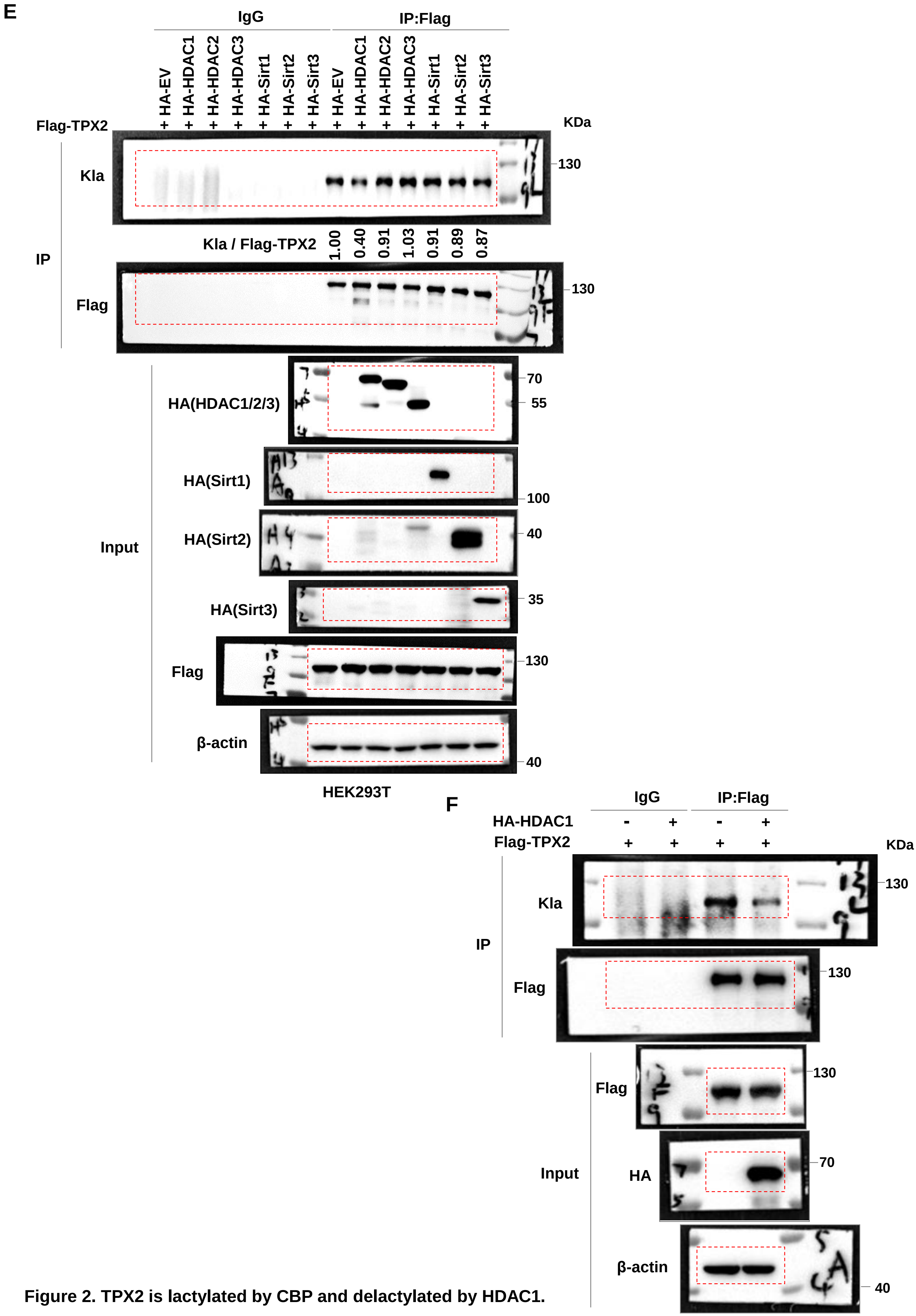

Figure 2. TPX2 is lactylated by CBP and delactylated by HDAC1.

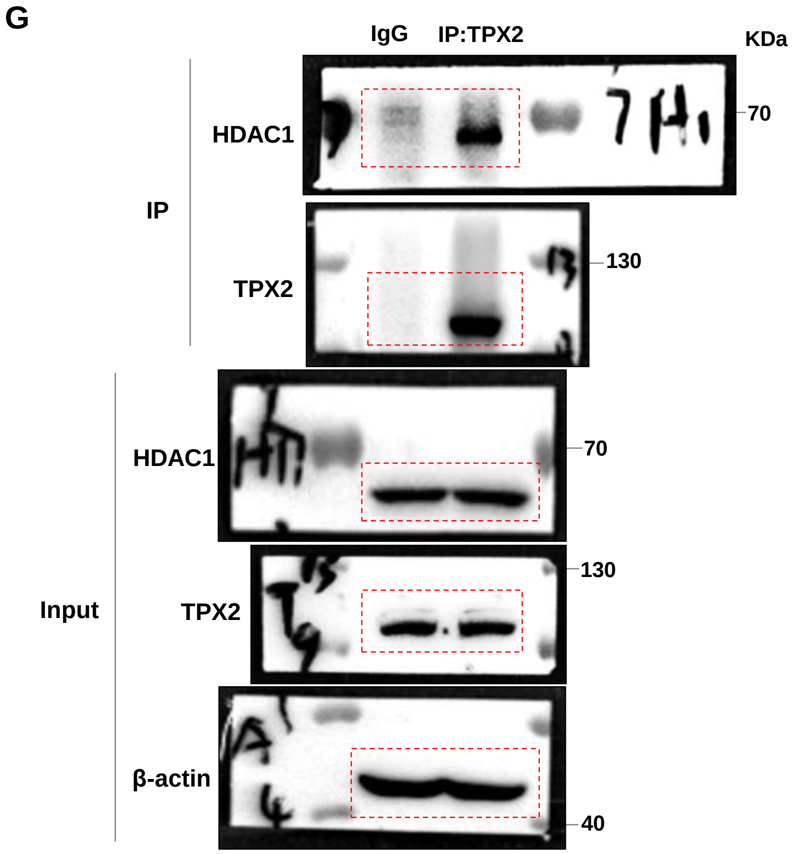

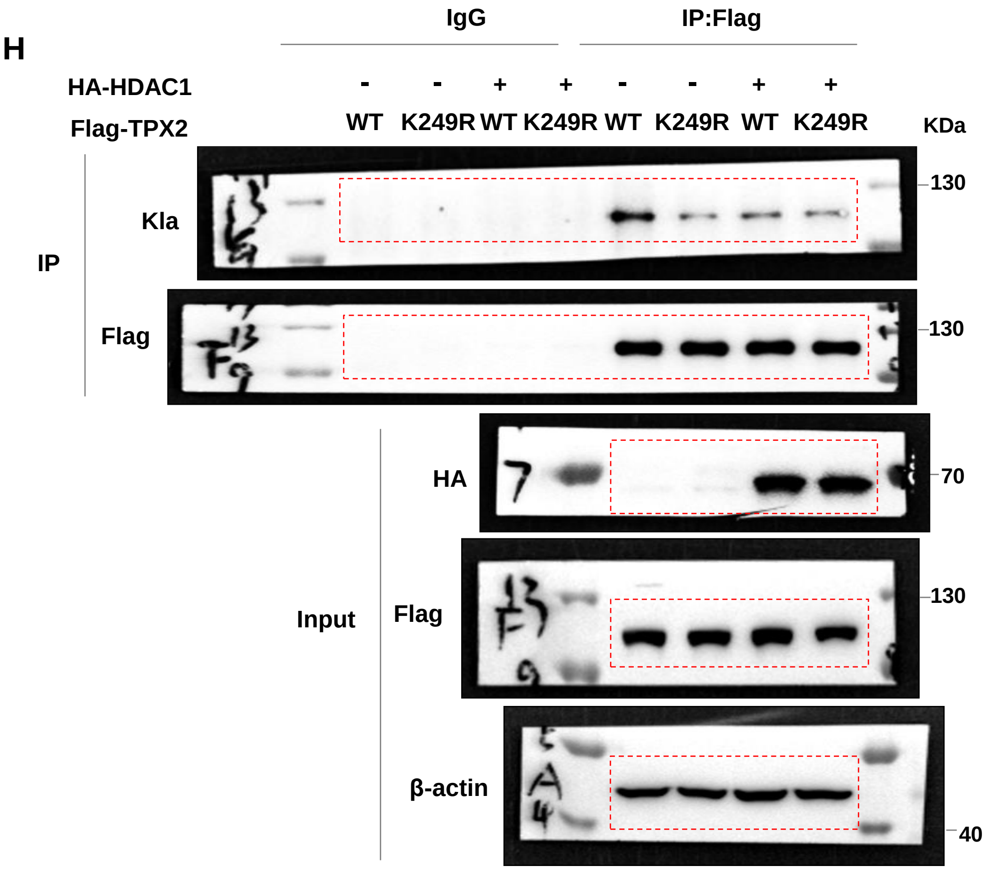

Figure 2. TPX2 is lactylated by CBP and delactylated by HDAC1.
