# Supplementary figures and images for: TPX2 lactylation is required for the cell cycle regulation and hepatocellular carcinoma progression
Source: Life Sci Alliance. 2025 Mar 19;8(6):e202402978. doi: 10.26508/lsa.202402978 (PMC11924114; doi:10.26508/lsa.202402978)

**A**

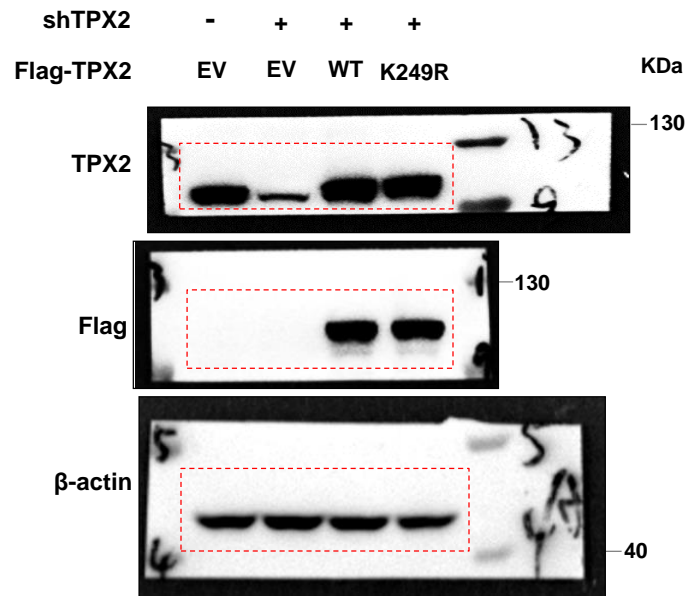

**F**

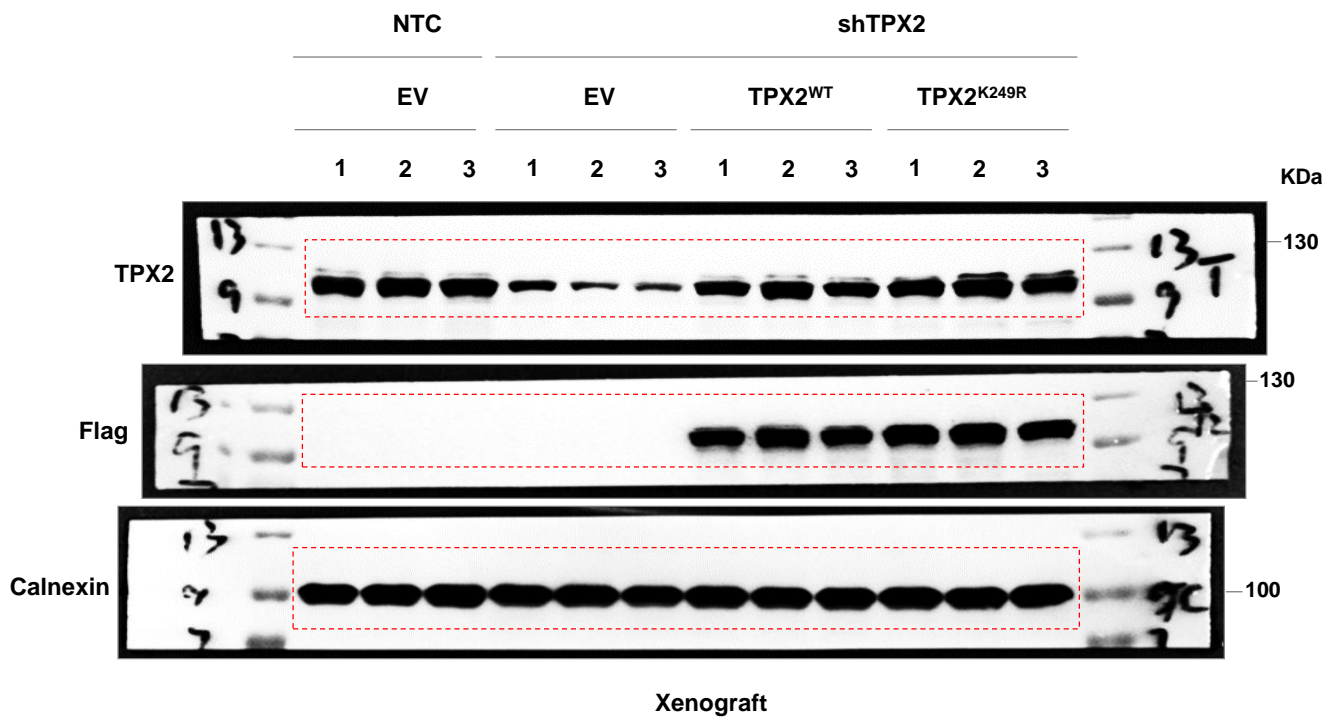

**Figure 3. TPX2 lactylation promotes tumour growth *in vitro* and *in vivo*.**

Supplement: Supplementary file 4 [file LSA-2024-02978_SdataF3.2.pdf]
