# Supplementary material for: TPX2 lactylation is required for the cell cycle regulation and hepatocellular carcinoma progression
Source: Life Sci Alliance. 2025 Mar 19;8(6):e202402978. doi: 10.26508/lsa.202402978 (PMC11924114; doi:10.26508/lsa.202402978)
Supplement: Supplementary file 5 [file LSA-2024-02978_SdataF4.2.pdf]

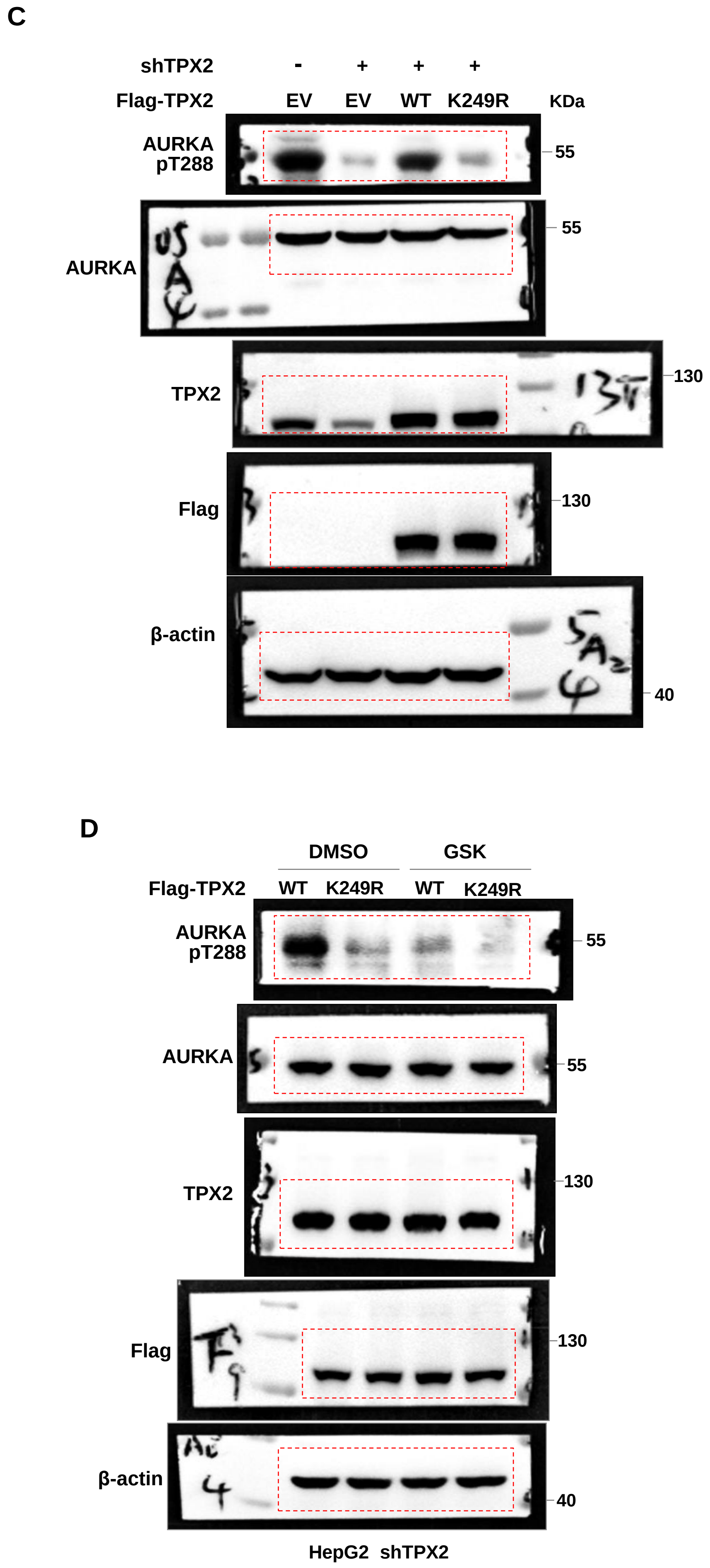

Figure 4. TPX2 lactylation is necessary for cell cycle progression by increasing AURKA phosphorylation.

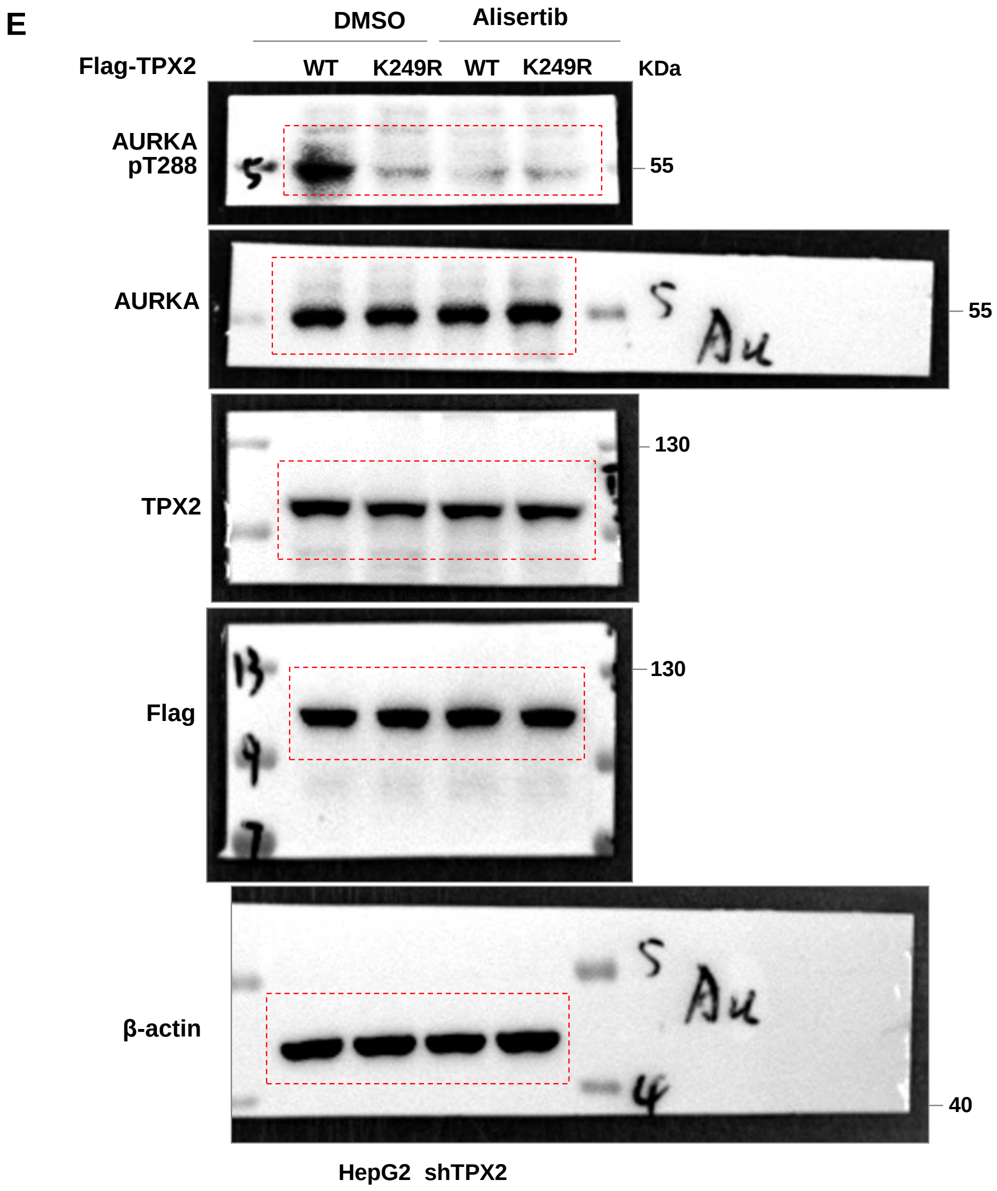

Figure 4. TPX2 lactylation is necessary for cell cycle progression by increasing AURKA phosphorylation.

H

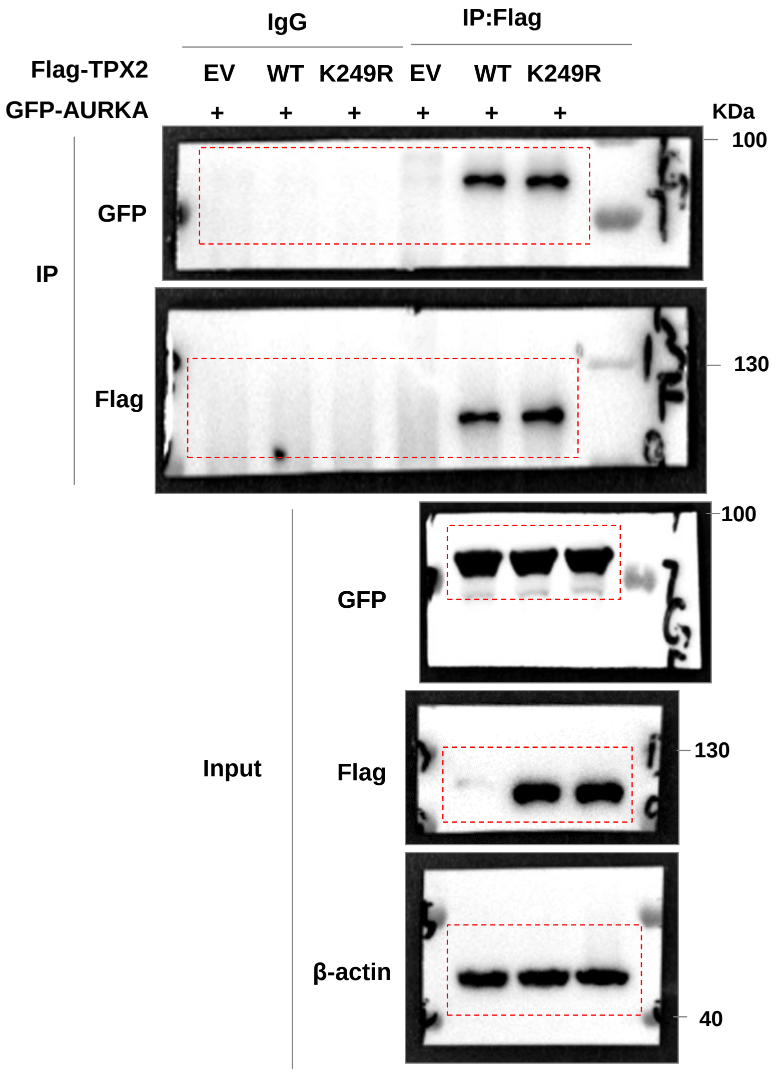

Figure 4. TPX2 lactylation is necessary for cell cycle progression by increasing AURKA phosphorylation.

I

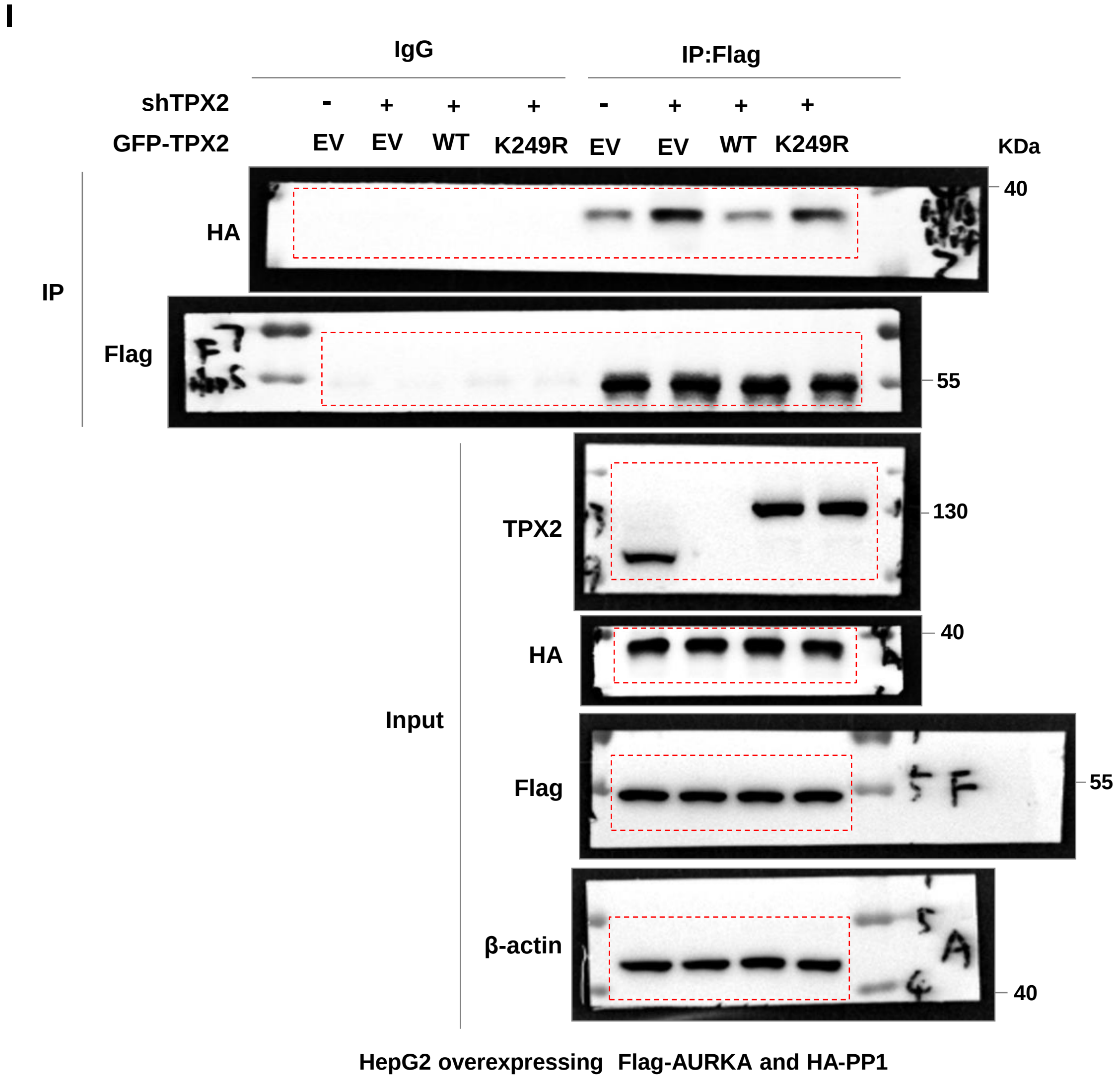

Figure 4. TPX2 lactylation is necessary for cell cycle progression by increasing AURKA phosphorylation.

J

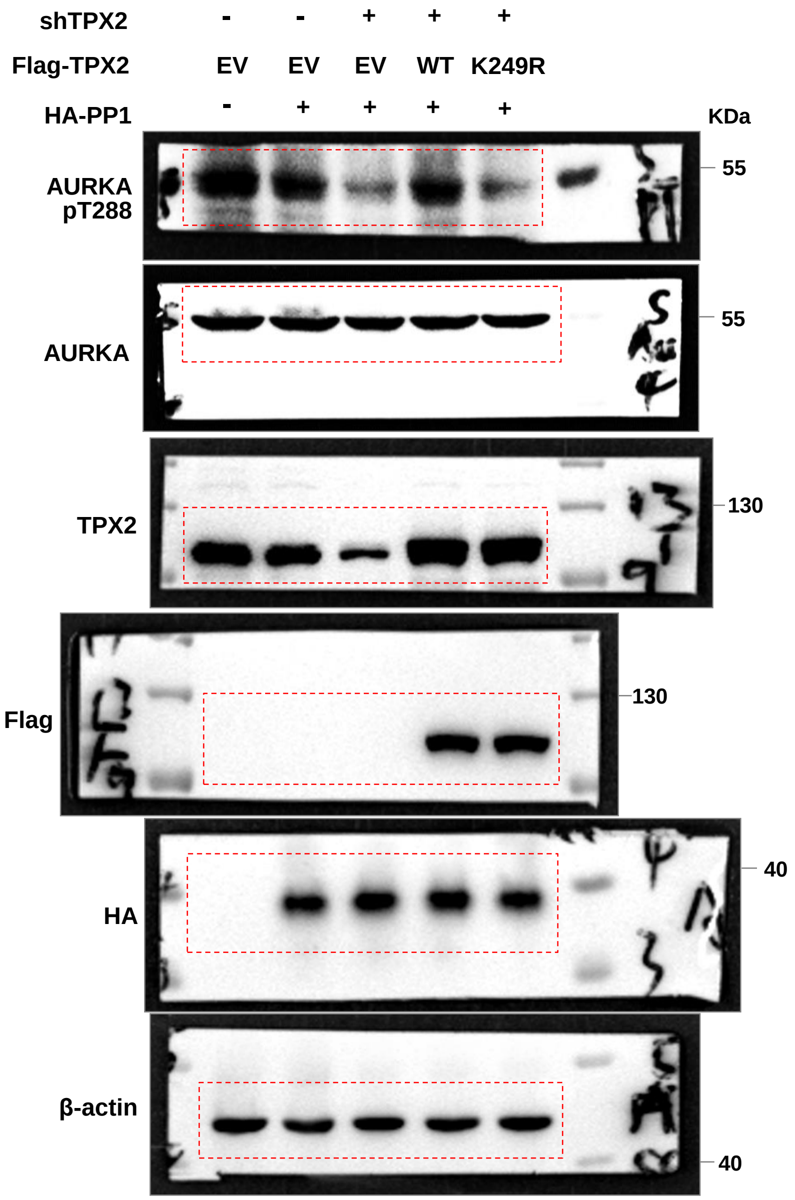

Figure 4. TPX2 lactylation is necessary for cell cycle progression by increasing AURKA phosphorylation.
