# Supplementary material for: TPX2 lactylation is required for the cell cycle regulation and hepatocellular carcinoma progression
Source: Life Sci Alliance. 2025 Mar 19;8(6):e202402978. doi: 10.26508/lsa.202402978 (PMC11924114; doi:10.26508/lsa.202402978)
Supplement: Supplementary file 6 [file LSA-2024-02978_TableS1.docx]

**Reagents and Tools Table**

| **Reagent/Resource** | **Reference or Source** | **Identifier or Catalog Number** |
| --- | --- | --- |
| **Experimental Models** |  |  |
| HepG2 cells (H. sapiens) | ATCC | HB-8065 |
| Hep3B cells (H. sapiens) | ATCC | HB-8064 |
| HEK293T (H. sapiens) | ATCC | CRL-3216 |
| BALB/c nude mice | Shanghai Laboratory Animal Center | N/A |
|  |  |  |
|  |  |  |
|  |  |  |
| **Recombinant DNA** |  |  |
| pSin-3×Flag-EV | This study |  |
| pSin-Flag-TPX2^WT^ | This study |  |
| pSin-Flag-TPX2^K249R^ | This study |  |
| pSin-HA-EV | This study |  |
| pSin-HA-P300 | This study |  |
| pSin-HA-CBP | This study |  |
| pSin-HA-KAT5 | This study |  |
| pSin-HA-KAT8 | This study |  |
| pSin-HA-GCN5 | This study |  |
| pSin-HA-HDAC1 | This study |  |
| pSin-HA-HDAC2 | This study |  |
| pSin-HA-HDAC3 | This study |  |
| pSin-HA-Sirt1 | This study |  |
| pSin-HA-Sirt2 | This study |  |
| pSin-HA-Sirt3 | This study |  |
| pSin-HA-TPX2 | This study |  |
| pSin-Flag-CBP | This study |  |
| pSin-Flag-HDAC1 | This study |  |
| pSin-GFP-AURKA | This study |  |
| pSin-GFP-TPX2^WT^ | This study |  |
| pSin-GFP-TPX2^K249R^ | This study |  |
| pSin-mCherry-TPX2^WT^ | This study |  |
| pSin-mCherry-TPX2^K249R^ | This study |  |
| pSin-Flag-AURKA | This study |  |
| pSin-HA-PP1（PPP1CA） | This study |  |
|  |  |  |
| **Antibodies** |  |  |
| Rabbit anti-TPX2 | Abcam | ab252945 |
| Rabbit anti-TPX2 | Proteintech | 11741-1-AP |
| Rabbit anti-Pan-Kla | PTMBIO | PTM-1401RM |
| Mouse anti-AURKA | PTMBIO | PTM-5308 |
| Rabbit anti-AURKA | Proteintech | 10297-1-AP |
| Rabbit anti-P-AURKA（T288） | Cell Signaling Technology | 3079T |
| Rabbit anti-LDHA | Proteintech | 21799-1-AP |
| Rabbit anti-CBP（CREBBP） | Proteintech | 22277-1-AP |
| Rabbit anti-CBP（CREBBP） | Cell Signaling Technology | 7389 |
| Rabbit anti-HDAC1 | Proteintech | 10197-1-AP |
| Rabbit anti-HDAC1 | Cell Signaling Technology | 34589T |
| Mouse anti-FLAG-M2 | Sigma-Aldrich | F1804 |
| Rabbit anti-HA | Proteintech | 51064-2-AP |
| Rabbit anti-GFP | Proteintech | 66002-1-Ig |
| Mouse anti-β-Actin | Proteintech | 66009-1-Ig |
| Rabbit anti-Calnexin | Proteintech | 10427-2-AP |
| Alexa Fluor® 488 Anti-alpha Tubulin | Abcam | ab195887 |
| HRP-conjugated anti-rabbit | Bio-Rad | Cat# 170-6515; |
| HRP-conjugated anti-mouse | Bio-Rad | Cat# 170-6516; |
| Mouse IgG1 isotype control Monoclonal antibody | Proteintech | 66360-1-Ig |
| Rabbit Anti-secondary antibodies conjugated to CoraLite594 | Abcam | ab150080 |
| Rabbit Anti-secondary antibodies conjugated to CoraLite555 | Proteintech | RGAR003 |
|  |  |  |
| **Oligonucleotides and other sequence-based reagents** |  |  |
| shRNA targeted sequence | This study | Table S1 |
|  |  |  |
|  |  |  |
| **Chemicals, Enzymes and other reagents** |  |  |
| Fetal Bovine Serum | Biological Industries | 04-001-1ACS |
| Medium Dulbecco's Modified Eagle Medium | Thermo Fisher Scientific | 12800-017 |
| HEPEs | Thermo Fisher Scientific | 15630-080 |
| Penicillin-Streptomycin Solution | Biological Industries | 03-031-1B |
| 0.25% Trypsin | Biological Industries | B03-050-1A |
| Opti-MEM | Thermo Fisher Scientific | 31985-088 |
| Polyethylenimine linear 25kD | Polyscience | 23966-2 |
| Polybrene | Sigma-Aldrich | H9268-5G |
| Puromycin | Sigma-Aldrich | P8833-100 mg |
| Cocktail | Sigma-Aldrich | 5056489001 |
| PMSF | Sangon Biotech | A610425-0005 |
| Triton X-100 | Sangon Biotech | A600198 |
| Protein A/G Beads | Thermo Fisher Scientific | 53133 |
| L-(+)-Lactic acid | Sigma-Aldrich | L6402 |
| Sodium oxamate | Sigma-Aldrich | O2751 |
| Dimethyl Sulfoxide | Sangon Biotech | A100231-0500 |
| Phosphatase Inhibitor Cocktail II | MCE | HY-K0022 |
| GSK2837808A | MCE | HY-100681 |
| Alisertib(MLN8237) | MCE | HY-10971 |
| CCK | APEXBIO | K1018 |
| Propidium Iodine | Sigma-Aldrich | P4170 |
| Rnase A | TIANGEN | RT405-02 |
| Ethanol | Sangon Biotech | A500737-0500 |
| T4 ligase | Thermo Fisher Scientific | 15224017 |
| Ligation-Free Cloning System | abmGood | E001-5-B |
| Ampicillin sodium | Sangon Biotech | A610028-0025 |
| Matrigel | Corning | 354230 |
|  |  |  |
| **Software** |  |  |
| GraphPad Prism 8 | https://www.graphpad.com/ |  |
|  |  |  |
|  |  |  |
| **Other** |  |  |
|  |  |  |
|  |  |  |
|  |  |  |
